# The feasibility of patient-reported outcomes, physical function, and mobilization in the care pathway for head and neck cancer surgical patients

**DOI:** 10.1186/s40814-022-01074-4

**Published:** 2022-05-27

**Authors:** Julia T. Daun, Rosie Twomey, Lauren C. Capozzi, Trafford Crump, George J. Francis, T. Wayne Matthews, Shamir Chandarana, Robert D. Hart, Christiaan Schrag, Jennifer Matthews, C. David McKenzie, Harold Lau, Joseph C. Dort, S. Nicole Culos-Reed

**Affiliations:** 1 Faculty of Kinesiology, University of Calgary, Calgary, AB Canada; 2 Ohlson Research Initiative, Arnie Charbonneau Cancer Institute, Cumming School of Medicine, University of Calgary, Calgary, AB Canada; 3 O’Brien Institute for Public Health, Cumming School of Medicine, University of Calgary, Calgary, AB Canada; 4 Department of Clinical Neurosciences, Cumming School of Medicine, University of Calgary, Calgary, AB Canada; 5 Department of Community Health Sciences, Cumming School of Medicine, University of Calgary, Calgary, AB Canada; 6 Department of Surgery, Cumming School of Medicine, University of Calgary, Calgary, AB Canada; 7 Department of Oncology, Cumming School of Medicine, University of Calgary, Calgary, AB Canada; 8 Section of Otolaryngology Head & Neck Surgery, Department of Surgery, Cumming School of Medicine, University of Calgary, Calgary, AB Canada; 9 Foothills Medical Centre, Alberta Health Services, Calgary, AB Canada; 10 Section of Plastic and Reconstructive Surgery, Department of Surgery, University of Calgary, Calgary, AB Canada; 11 Department of Psychosocial Resources, Tom Baker Cancer Centre, Cancer Care, Alberta Health Services, Calgary, AB Canada

**Keywords:** Head and neck cancer, Exercise oncology, Prehabilitation, Feasibility

## Abstract

**Background:**

Head and neck cancer (HNC) patients are an understudied population whose treatment often includes surgery, causing a wide range of side effects. Exercise prehabilitation is a promising tool to optimize patient outcomes and may confer additional benefits as a prehabilitation tool. The primary objective of this study was to assess the feasibility of measuring patient-reported outcomes (PROs), physical function, and in-hospital mobilization across the HNC surgical timeline in advance of a future prehabilitation trial. The secondary objective was to examine potential changes in these outcomes across the surgical timeline.

**Methods:**

HNC patients scheduled to undergo oncologic resection with free-flap reconstruction completed assessments of PROs and physical function at three timepoints across the surgical timeline (baseline, in-hospital, and postsurgical/outpatient). Mobilization was measured during the in-hospital period. The feasibility of recruitment and measurement completion was tracked, as were changes in both PROs and physical function.

**Results:**

Of 48 eligible patients, 16 enrolled (recruitment rate of 33%). The baseline and in-hospital PROs were completed by 88% of participants, while the outpatient assessments were completed by 81% of participants. The baseline and in-hospital assessment of physical function were completed by 56% of participants, and 38% completed the outpatient assessment. Measuring in-hospital mobilization was completed for 63% of participants.

**Conclusion:**

Measuring PROs and in-hospital mobilization is feasible across the surgical timeline in HNC; however, the in-person assessment of physical function prior to surgery was not feasible. A multidisciplinary collaboration between exercise specialists and clinicians supported the development of new clinical workflows in HNC surgical care that will aid in the implementation of a future prehabilitation trial for this patient population.

## Key messages regarding feasibility

- **What uncertainties existed regarding the feasibility?**
  Considering the unique nature of the HNC surgical trajectory, in advance of conducting an exercise prehabilitation clinical trial, it is necessary to establish whether it is feasible to recruit patients before surgery and if the timing and type of assessments required to evaluate exercise prehabilitation are feasible.
- **What are the key feasibility findings?**
  Measuring PROs and in-hospital mobilization was feasible across the surgical timeline in HNC. The in-person assessment of physical function prior to surgery was not feasible.
- **What are the implications of the feasibility findings for the design of the main study?**
  The current findings are supporting the development of an exercise prehabilitation trial in HNC surgical patients — one that does not include an in-person assessment prior to surgery, includes home-based exercise support prior to surgery, and supports patients with inpatient and online resources across the surgical timeline.

## Background

Head and neck cancer (HNC) is the 7th most commonly diagnosed cancer worldwide [1]. Treatment for HNC is multimodal, with the majority of patients undergoing surgery [2]. Surgical treatment often leads to side effects that potentially impair both physical and psychosocial functioning [3, 4]. While patient care over the perioperative period has improved, including implementation of enhanced recovery after surgery (ERAS) protocols to optimize intra- and postoperative care, gaps in preoperative care and associated patient outcomes remain [5, 6]. One means to enhance patient care and improve outcomes is exercise prehabilitation programs [7].

Exercise prehabilitation is an intervention designed to take place prior to surgery to optimize physical and psychosocial functioning, improving surgical tolerability and facilitating recovery [8, 9]. This prehabilitation period also presents an opportunity to provide patients with individualized care, decreased treatment-related morbidity, and increased sense of control [8, 9]. Exercise prehabilitation in a number of other tumor groups (i.e., lung, pancreatic, colorectal, breast) shows improved physical function prior to surgery [10, 11], but this has yet to be examined in HNC. Beyond prehabilitation, exercise is a tool used widely in oncology to improve treatment outcomes and overall patient health status [12]. Research examining the role of exercise for HNC patients during and after adjuvant treatment concludes that exercise is safe, feasible, and beneficial for physical functioning, quality of life (QOL), and symptom management [13, 14].

Considering HNC surgical patients comprise a unique patient population, it is necessary to establish the role of exercise earlier in the HNC treatment pathway. Answering questions around the feasibility of recruiting patients before surgery, as well as the timing and type of assessments required to evaluate exercise prehabilitation, are foundational components of work that are important in advance of time- and resource-intensive exercise prehabilitation clinical trials [15].

The primary objective of this feasibility study was thus to assess the feasibility of measuring patient-reported outcomes (PROs), physical function, and in-hospital mobilization across the HNC surgical timeline. The secondary objective was to examine potential changes in these outcomes across the surgical timeline. This work will inform subsequent development of the components of an exercise prehabilitation intervention in HNC patients.

## Methods

### Participants

This study was approved by the University of Calgary Health Research Ethics Board of Alberta (HREBA) — cancer committee (CC) — HREBA.CC-18-0564. All HNC patients scheduled to undergo oncologic resection for benign or malignant disease with free-flap reconstruction were invited to participate. Exclusion criteria included age < 18 years, inability to read/write in English, low cognitive function, and inability to participate in the tests of physical function. As a non-interventional feasibility study, no a priori sample size was calculated. Based on current clinical numbers, we anticipated six eligible patients per month over a recruitment period of 10 months. Thus, total potential sample size was 60.

### Study design and procedure

This feasibility study recruited HNC patients attending a surgical consult in Calgary, Canada, with those eligible for the study identified by a member of the clinical team (i.e., surgeon or oncology nurse), who obtained a “consent to contact.” Contact details were provided to the study coordinator, and the patient was contacted within 72 business hours. Those who agreed to participate were sent a link to the informed consent process via a secure web application (Research Electronic Data Capture; REDCap) [16]. The introduction of the study both in clinic and during the initial phone call was standardized using a script. Reasons for not participating were recorded.

Consenting patients completed assessments at three timepoints: (1) baseline, approximately 72 business hours after surgical consent; (2) 10–14 days post-surgery, as an inpatient; and (3) 6 ± 2 weeks post-surgery, as an outpatient. Of note, the average time from surgical consent to surgery in Calgary is 25 ± 19 days for HNC surgical patients. Assessments included PROs, measures of physical function, and in-hospital mobilization measured via a wearable activity tracker. Reasons for participants unable to complete a specific measure or time point were recorded.

#### Baseline

Participants completed baseline PROs via REDCap. An in-person assessment of physical function (60-min duration) was scheduled at the Health and Wellness Lab, University of Calgary. In some cases, this assessment was scheduled at the location of the next clinical appointment (i.e., due to travel and time constraints).

#### In-hospital

A postsurgical inpatient assessment took place shortly before hospital discharge (10–14 days after surgery) on the unit. A member of the research team collected PROs via REDCap using an iPad and the same tests of physical function as baseline.

#### Outpatient

A postsurgical*,* outpatient assessment of PROs and physical function was set for 6 ± 2 weeks after surgery. This was pre-adjuvant treatment for those scheduled to receive it.

### Demographics and clinical characteristics

Sociodemographic and lifestyle data, including self-reported race/ethnicity, gender, employment status, annual family income, smoking status, and alcohol consumption, was collected via REDCap. Clinical characteristics were collected via chart review and included sex, clinical diagnosis (tumor site, cancer stage), and treatment protocol (i.e., surgery only or surgery and adjuvant therapy). Age was collected in REDCap and confirmed with chart review.

### Primary outcome: assessment of feasibility

#### Recruitment

The criteria for feasibility for overall recruitment rate was predetermined as ≥ 70%. Recruitment rate was defined as the number of enrolled patients from the total number of eligible patients. In order to further understand the overall recruitment rate, two sub-components were tracked: (1) the number of referrals from the clinical team (referral rate) and (2) the number of patients who agreed to participate once they heard a full introduction of the study (enrolment rate).

#### Measurement completion

Measurement completion rate, defined as completion of PROs, tests of physical function, and assessment of early mobilization (i.e., number of participants wearing the activity tracker during the in-hospital period), was recorded at each assessment timepoint. The criteria for feasibility of measurement completion was predetermined as a measurement completion rate of ≥ 60% at each timepoint.

#### Adverse events

To assess safety of this feasibility study, all adverse events were tracked. The feasibility study was determined safe if no major adverse events occurred.

### Secondary outcome: trends in PROs and physical function over time

#### Patient-reported outcomes

PROs were chosen based on their established validity, previous use in HNC patients, and relevance for use in the future intervention study, to evaluate the benefits of exercise prehabilitation.

##### Quality of life

To measure QOL specific to HNC, the Functional Assessment of Cancer Therapy-Head and Neck (FACT-H&N) was used [17, 18]. It includes five subscales: (1) physical well-being, (2) social/family wellbeing, (3) emotional wellbeing, (4) functional wellbeing, and (5) additional (HNC-specific) concerns, which are summed to create a total score [17, 18]. In addition, the FACE-Q module for HNC was used [19]. This questionnaire includes standalone sub-scales that quantify domains including QOL, experience of care, appearance, eating and drinking, oral competence, salivation, smiling, speaking, and swallowing.

##### General health status

To measure health status, the EuroQol visual analogue scale (EQ-5D VAS), an internationally recognized and generic measure of health, was used [20]. The EQ-5D VAS is a 20-cm vertical visual analogue scale (VAS) where respondents can indicate their self-rated health, ranging from the best to the worst health states they can imagine.

##### *Depression and anxiety*

Depression and anxiety were assessed using the Hospital Anxiety and Depression Scale (HADS) [21] which has previously been used in HNC patients and has demonstrated high screening accuracy for this population [22].

##### *Symptom burden*

Symptom burden was assessed using the revised Edmonton Symptom Assessment System (ESAS-r), which evaluates nine common symptoms experienced by cancer patients [23, 24]. The ESAS-r is part of the standard care pathway at the Tom Baker Cancer Centre in Calgary, Alberta, and is part of the *putting patients first* tool used in cancer care (Alberta Health Services).

##### Fatigue

Fatigue was measured using the Functional Assessment of Chronic Illness Therapy Fatigue (FACIT-F) scale [25]. This questionnaire is validated and commonly used as a measure of fatigue in cancer patients [26].

##### *Physical activity*

Self-reported physical activity levels were assessed using a modified Godin Leisure-Time Exercise Questionnaire (GLTEQ) [27]. This questionnaire asks participants to recall their average weekly exercise over the past month, including the frequency and duration of mild, moderate, and strenuous aerobic activity, as well as frequency and duration of resistance and flexibility exercise. Summing the scores provides an overall exercise level, which can be compared to current cancer survivor guideline recommendations [28].

#### Physical function

The assessment of physical function was conducted in the order described below. During the assessment, participants wore grip socks in order to replicate hospital conditions. For the participants who preferred to wear shoes, this was replicated across all timepoints.

##### *Body composition*

Only for those attending an in-person assessment at the Health and Wellness Lab, body composition was measured at baseline and at the outpatient assessments. Height was measured using a seca 217 stadiometer. Weight was measured using a 16 Health Carter Beam Scale. A dual-energy x-ray absorptiometry (DXA) scan (Hologic QDR 4500; Hologic, Inc, Bedford, MA) was used to obtain total lean mass and total fat mass.

##### *Handgrip strength*

Handgrip strength is associated with the risk of adverse health outcomes [29] and provides a valid marker of overall muscular strength [30]. This test is conducted using a hand dynamometer and followed the Canadian physical activity, fitness & lifestyle approach (CPAFLA) protocol [31]. Due to the use of the forearm as a donor site, not all participants could perform this measure on both sides. Therefore, scores from the dominant or non-donor hand were used in analyses.

##### *Static balance ability*

The timed single-leg stance measures static balance ability [32]. The protocol followed the Canadian Society for Exercise Physiology (CSEP) guidelines [33]. The test was conducted twice: first with eyes open and second with eyes closed, for a maximum of 45 s on each leg. The best score of two trials was recorded for each condition [33], but depending on the location of a participant’s donor site, scores from the dominant or non-donor leg were used in analyses.

##### *Lower limb muscular endurance*

The 30-s sit-to-stand (the number of full stands from a seated position in 30 s) measures lower limb muscular endurance and has been previously used in HNC patients [34], with good test-retest reliability [35]. The same chair (height ~46 cm) was used both in the exercise laboratory and in-hospital.

##### *Functional exercise capacity*

The 6-min walk test (6MWT) assesses functional exercise capacity and has established measurement properties [36], including validity for use in cancer patients, including HNC [35]. The 6MWT was performed following the American Thoracic Society (ATS) protocol [37] using a 20-m hallway and standardized instructions.

#### Mobilization

To measure in-hospital mobilization, a member of the clinical team provided participants with a wearable activity tracker, the Garmin vivofit 4. This tracker has been used in previous studies to measure objective levels of physical activity [38]. The activity tracker was provided to participants 24–48 h after surgery and was worn continuously (i.e., kept on for 24 h per day) either on their wrist or ankle, depending on the free-flap donor site, up until their date of discharge. Specifically, participants’ daily step count was tracked. Based on clinical judgement and surgical recovery, participants were encouraged to increase their mobilization throughout their hospital stay. The clinical team was encouraged to track participants’ steps daily using a laminated mobilization tracking sheet and whiteboard marker. While recording mobilization on the tracking sheet was encouraged, it was not protocolized nor tracked.

### Data analysis

To assess feasibility, data was analyzed using descriptive statistics. Descriptive characteristics of the participants (Table 1), PROs (Table 2), and physical function (Table 3) are presented as mean ± standard deviation or percentages. For in-hospital mobilization, days with incomplete step data, (i.e., days where a participant did not wear the tracker for the full day, such as the day of discharge) were excluded from analysis, as done in previous studies that have tracked objective activity data [39]. These data are presented as mean ± standard deviation. Where available, the minimum clinically important difference (MCID) [40] was reported.

**Table 1:** Baseline characteristics and demographics of patients scheduled for oncologic resection with free-flap reconstruction, *n* = 16

Participant characteristics	No. of patients
Sex	
Male	13 (81%)
Female	3 (19%)
Age: mean ± SD, y	59.9 ± 8.2 (range: 44–71)
Time from surgical consult until surgery: mean ± SD, days	11.2 ± 7.7
Primary tumor site	
Oral cavity	13 (81%)
Oropharynx	2 (13%)
Paranasal sinuses	1 (6%)
Cancer stage	
I	1 (6%)
II	3 (19%)
III	3 (19%)
IV	6 (38%)
Unknown	3 (19%)
Histology: squamous cell carcinoma	16 (100%)
Treatment	
Surgery alone	7 (44%)
Surgery + radiation therapy	5 (31%)
Surgery + radiation therapy and chemotherapy	4 (25%)
Demographic variable	No. of patients
Race	
White	13 (81%)
Not specified	3 (19%)
Employment status	
Disability	1 (6%)
Part time	3 (19%)
Full time	8 (50%)
Unemployed	2 (13%)
Unknown	2 (13%)
Annual family income, CDN$	
$60,000–79,999	3 (19%)
$80,000–99,000	1 (6%)
> 100,000	4 (25%)
Prefer not to answer	6 (38%)
Unknown	2 (13%)
Smoking status	
Never smoked	4 (25%)
Ex-smoker	9 (56%)
Current smoker	3 (19%)
Alcohol consumption	
Never drinker	5 (31%)
Light drinker	3 (19%)
Moderate drinker	4 (25%)
Heavy drinker	2 (13%)
Previous drinker	2 (13%)

**Table 2:** Patient-reported outcomes at baseline, 7–15 days post-surgery and 6 ± 2 weeks post-surgery

Outcome measure	Baseline Pre-surgery (***n*** = 14)	7–15 days post-surgery (***n*** = 14)	6 ± 2 weeks Post-surgery (***n*** = 13)
**FACT-H&N**			
Physical well-being	24 ± 5	19 ± 5	23 ± 4
Social well-being	23 ± 7	23 ± 6	24 ± 3
Emotional well-being	18 ± 5	20 ± 3	20 ± 3
Functional well-being	20 ± 7	10 ± 4	17 ± 6
FACT-G total	**85 ± 19**	**71 ± 12**	**84 ± 17**
FACT-H&N additional concerns	36 ± 8	26 ± 4	31 ± 9
FACT-H&N total	121 ± 24	97 ± 14	114 ± 24
**FACIT-F**	**44 ± 9**	**26 ± 11**	**41 ± 9**
**EQ-5D VAS**	**71 ± 22**	**50 ± 23**	72 ± 21
**ESAS-r**	**15 ± 11**	**28 ± 13**	**19 ± 16**
**HADS anxiety**	**4 ± 3**	**5 ± 3**	3 ± 4
**HADS depression**	**3 ± 4**	**6 ± 4**	**4 ± 4**
**GLTEQ**			
Total PA score	30 ± 25		29 ± 41
Moderate and vigorous PA score	17 ± 19		17 ± 32
Number of RT sessions/week	0 ± 1		1 ± 2
Duration of RT sessions/week (mins)	13 ± 22		8 ± 11
Number of FT sessions/week	1 ± 2		3 ± 3
Duration of FT sessions/week (mins)	7 ± 8		11 ± 10

*FACT-H&N*, Functional Assessment of Cancer Therapy; *FACIT-F*, Functional Assessment of Chronic Illness Therapy; *ESAS-r*, Edmonton Symptom Assessment System (revised version); *GLTEQ*, Godin Leisure-Time Exercise Questionnaire; *HADS*, Hospital Anxiety and Depression Scale; *PA*, physical activity; *RT*, resistance training; *FT*, flexibility training. Values in **bold** depict clinically relevant changes in outcomes, as reported above

**Table 3:** Physical function at baseline, 7–15 days post-surgery and 6 ± 2 weeks post-surgery

Outcome measure	Baseline Pre-surgery ***n*** = 9	7–15 days Post-surgery ***n*** = 7	6 ± 2 weeks Post-surgery ***n*** = 6
Anthropometric variable			
Body mass (kg)	**103.7 ± 25.1**		**95.8 ± 27.0**
Height (cm)	175.8 ± 9.2		176. 5 ± 10.8
BMI, kg/m^2^	33.5 ± 8.1		30.7 ± 8.5
Lean body mass, kg	64.7		60.5
Body fat %	30.2		30.4
Grip strength	**48.6 ± 11.6**	**45.6 ± 11.7**	**47.8 ± 13.8**
Single-leg stance — eyes open	31.1 ± 16.6	30.9 ± 17.3	23.8 ± 17.0
Single-leg stance — eyes closed	6.8 ± 8.8	4.5 ± 2.1	11.4 ± 9.6
30-s sit to stand	**16.6 ± 5.4**	**12.8 ± 4.3**	18.2 ± 6.0
6MWT (m)	**496 ± 81**	**339 ± 138**	504 ± 140

*BMI*, body mass index; *6MWT*, 6-min walk test. Values in **bold** depict clinically relevant changes in outcomes, as reported above

## Results

### Participant characteristics and demographics

Table 1 presents the clinical characteristics and demographics of study participants. The majority of patients enrolled were male (81%), and age ranged from 44 to 71 years.

### Primary outcome: assessment of feasibility

#### Recruitment

**Fig. 1:**
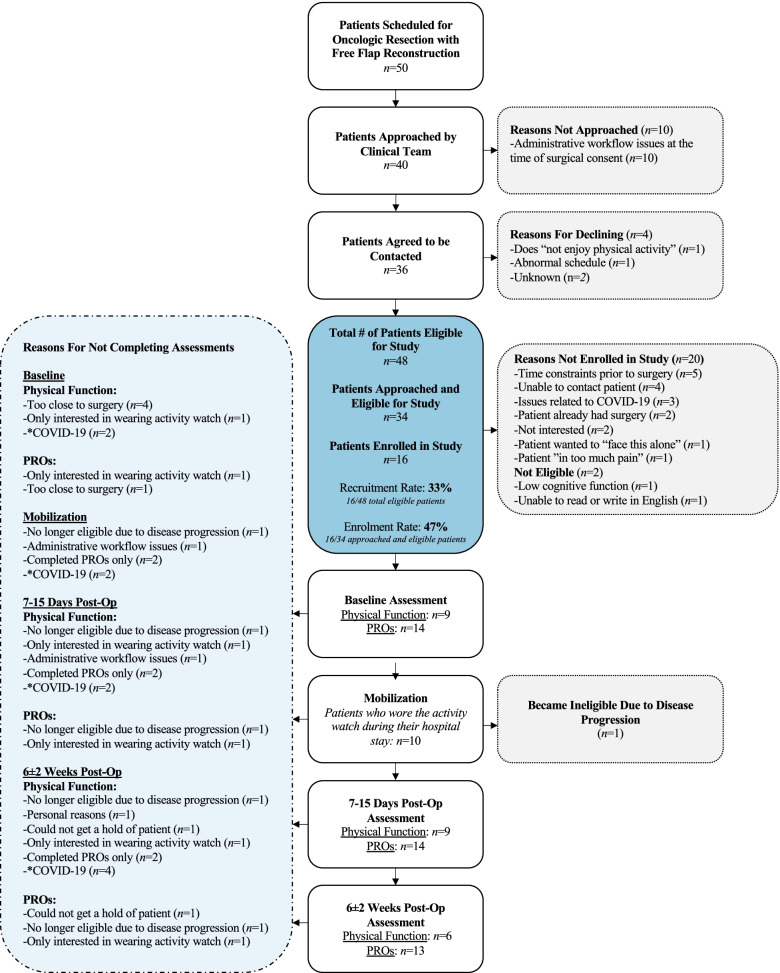
Participant flowchart indicating feasibility of recruitment, enrolment, and assessments. PROs, physical function. PROs, patient-reported outcomes; post-op, postoperative; *disruption to study due to the COVID-19 pandemic

The recruitment flow chart is depicted in Fig. 1. Recruitment occurred over an 8-month period between August 2019 and March 2020, closing 2 months earlier than intended due to the COVID-19 pandemic. During recruitment, 50 patients were scheduled for oncologic resection with free-flap reconstruction, of which 40 (80%) were introduced to the study by a member of the clinical team, via the consent to contact process. The remaining ten patients (20%) were not approached due to administrative workflow issues at the time of surgical consent.

Of the 40 patients who completed consent to contacts, four declined to be contacted, and two were deemed ineligible for the study after the initial recruitment phone call. Of the 34 eligible patients who heard the full introduction of the study, 16 enrolled into the study for an enrolment rate of 47% (i.e., number of patients who agreed to participate after hearing the fully study introduction). All reasons for patients not enrolling are present in Fig. 1. The main were (1) time constraints prior to surgery (*n* = 5) and (2) inability to contact the patients (*n* = 4). In addition, there were five patients who were not enrolled due to other circumstances, including contact after their surgery due to an administrative error at the clinic (*n* = 2) and disruption due to the COVID-19 pandemic (*n* = 3). The overall recruitment rate was 33% (i.e., the number of patients enrolled from overall eligible patients even if they were not approached by the clinical team 16/48). The referral rate (i.e., the number of referrals from the clinical team) increased from 22% of potentially eligible patients in the first month of recruitment to 100% of eligible patients in the final month of recruitment.

#### Measurement completion

##### *Patient-reported outcomes*

The completion rate for PRO questionnaires across all timepoints (i.e., baseline, in-hospital, and outpatient) was 88%, 88%, and 81%, respectively. The main reason for not completing PROs prior to surgery was time constraints (*n* = 2; Fig. 1). For the in-hospital and outpatient assessments, two participants did not complete PROs; one became ineligible (disease progression), and the other chose to only use the activity tracker and not complete any further measures.

##### *Physical function*

Nine participants completed the baseline assessment, nine completed the in-hospital assessment, and six completed the outpatient assessment (*note*: not all of the same participants completed all timepoints, with five completing assessments at all three points). The baseline assessment took place between 4 h and 8 days after initial phone call and was scheduled based on availability given the time available prior to surgery. The in-hospital assessment was between 7 and 15 days after surgery (mean days 10 ± 3). The outpatient assessment took place between 5 and 8 weeks after surgery (6.8 ± 1.2). The completion rate for these assessments was 56%, 56%, and 38%, respectively. The main reason for not completing measures of physical function included time constraints prior to surgery, personal reasons, and the COVID-19 pandemic (Fig. 1).

##### *Mobilization*

**Fig. 2:**
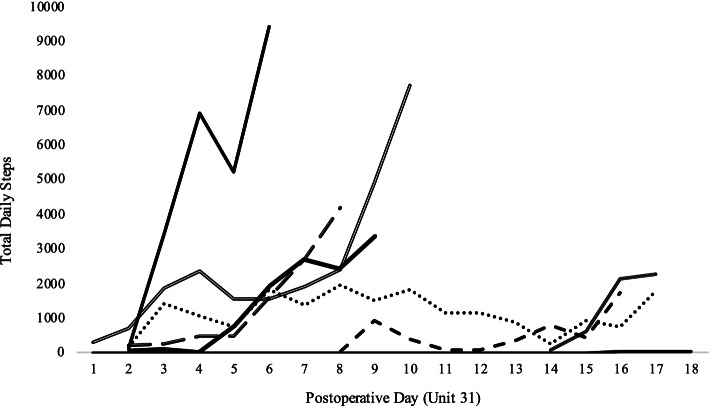
Total daily steps across the in-hospital period

During the in-hospital period, 10 participants (63%) wore the activity tracker. Of these participants, five had complete data sets (i.e., from postoperative day 1 or 2 to discharge), and five had partial data collected (i.e., between 2 and all days of hospital stay). The main reasons for partial data collected included post-operative complications (e.g., participant had a stroke), interruptions in clinical workflow resulting in the participants not receiving the activity tracker as planned, or technical issues with the activity tracker (e.g., syncing issues). Two participants did not wear the activity tracker because nonessential patient interaction was halted due to COVID-19. Figure 2 presents the change in step count during hospital stay for participants with ≥ 4 days of consecutive data collection (*n* = 7). All participants had a graded increase in steps across the hospital stay, ranging from 32–323 steps on days 1 or 2 post-surgery to 1736–9436 steps on the day before discharge.

#### Adverse events

No major or minor adverse events occurred.

### Secondary outcomes: trends in PROs and physical function over time

#### Patient-reported outcomes

As presented in Table 2, the decrease in QOL (FACT-H&N total score) from pre-surgery to in-hospital was clinically relevant (*MCID* = 6–12 point s[41]) and did not return to baseline levels at the outpatient assessment. Ratings of health status (EQ-5D VAS) decreased by more than the MCID (7–10 [42]) but tended to return to baseline levels by the outpatient assessment. Symptoms of depression and anxiety increased by more than the MCID from pre-surgery to in-hospital (*MCID* = 1.5 [43]), and only anxiety levels decreased by the outpatient assessment. The increases in symptom burden (ESAS-r total) and fatigue (FACTI-F) during the in-hospital period were clinically meaningful (*MCIDs* = 1 [44] and 3–6 [25], respectfully), and scores did not return to baseline levels by the outpatient assessment. Finally, overall self-reported physical activity levels did not appear to differ between the baseline to outpatient assessment. A table containing FACE-Q data is available online (https://osf.io/qw4zx).

##### *Physical function*

Descriptive analyses of body composition and physical function are presented in Table 3. Body mass index (BMI) across all participants decreased from the baseline to the outpatient assessment, presenting a clinically meaningful change (*MCID* = 5–10% [45]). As presented in Table 3, muscular strength decreased from pre-surgery to in-hospital and did not return to pre-surgery levels at the outpatient assessment. Both of these changes were clinically meaningful (*MCID* = 2.69 [46]). Single-leg stance (eyes open) in the dominant or non-donor leg tended to decrease over time, but changes were not clinically relevant (*MCID* = 24 s [47]). Muscular endurance (30-s sit to stand) decreased between the baseline and in-hospital assessment but tended to increase by the outpatient assessment, and both changes were clinically relevant (*MCID* = 10% [48]). Lastly, distance covered in the 6MWT was substantially reduced from the baseline to in-hospital assessment (by 157 ± 57 m, where the *MCID* = 31 m [49]) but tended to return to baseline values at the outpatient assessment.

## Discussion

The primary objective of this study was to assess the feasibility of measuring PROs, physical function, and in-hospital mobilization across the HNC surgical timeline. The secondary objective was to examine potential changes in these outcomes. As a unique patient population, such preliminary work is necessary to establish whether it is feasible to recruit patients before surgery and if the timing and type of assessments required to evaluate exercise prehabilitation are feasible.

### Feasibility of recruitment

The overall recruitment rate of 33% was lower than the feasibility criteria (≥ 70%). However, an improvement in the referral rate (one of the subcomponents of the overall recruitment rate) between the first and final month of recruitment, from 22 to 100% referral, demonstrates the success in adoption of new clinical practices and workflow procedures to facilitate recruitment. Specifically, clinical workflows were optimized to ensure the success of the consent to contact process, including (1) regular reminders to the clinical team through biweekly e-mails and monthly meetings, (2) ensuring appropriate and sufficient study documentation (i.e., consent to contact forms, checklist for clinical team to refer to) at each clinical site, and (3) prospective review of appointments with potentially eligible patients. These results suggest that in our local clinical setting, patients can be referred by the clinical team at the time of surgical consult, to maximize the time available for a preoperative exercise intervention. It is also important to note that the predetermined feasibility criteria of ≥ 70% may have been set too high, where ≥ 50% may be more reasonable, as seen across other feasibility work in exercise oncology [50–52].

Overall, the number of patients enrolling into the study after the consent to contact process (47%) was also not feasible. The low enrolment rate was possibly due to a combination of time constraints prior to surgery, the need for an in-person assessment, and the lack of intervention that would result in personal benefits to the patients. However, with changes in workflow, enrolment increased to 83% by the end of the study.

### Feasibility of measurement completion

At all timepoints, PRO completion was above 80% and thus deemed feasible. This may be related to the ability to complete PROs online, from home. Adding PRO measurement to the surgical timeline is a critical component of patient-centered care and is necessary to increase the understanding and impact of a future exercise prehabilitation intervention [53].

At all timepoints, the completion rate for the measurements of physical function was lower than the predetermined feasibility level of 60%. The in-person baseline assessment was feasible for half (56%) of participants. Constraints included having multiple appointments, the stress pre-surgery, and being unable or unwilling to add in another assessment and unable to attend due to living out of town.

Wearing the activity tracker was feasible for 63% of participants. While we did not obtain full data sets for all participants, important lessons were learned for adopting new workflow and measurement of mobilization via step counts within a clinical care setting. First, regular communication with nursing staff (e.g., phone call reminders on postoperative day 1) ensured the delivery of the activity tracker to participants. Communication included (1) pre-study nursing round presentations, (2) e-mails and phone calls (i.e., when a participant was arriving on the unit, ensuring activity tracker was on participant, scheduling the inpatient assessment), (3) a study binder containing all details related to the study, and (4) a checklist for nurses to refer to for activity tracker administration and usage. Second, the activity tracker placement must be considered in relation to donor site to ensure accurate recording. Because some patients use a walking aid or walk with an intravenous pole after surgery, a strap extender can be utilized to move the device from wrist to ankle. Third, maintaining education on how and why to use the activity tracker is important for achieving participant engagement. Wearable technology is widely used in the area of oncology [54] and has previously been used in surgical settings within ERAS protocols [55]. These results highlight the potential for the activity tracker to be feasible and an important component for assessing in-hospital mobilization.

### Implications for a future exercise prehabilitation intervention

Since the aim of prehabilitation is to optimize patient outcomes prior to surgical treatment, and provide the patient with increased sense of control during an overwhelming time [56], it is of interest to utilize this period of time as effectively as possible. A key consideration with HNC surgical patients is the short timeframe between surgical consent and surgery (11.2 ± 7.7 days for our cohort of participants at our local setting), which has implication for what an effective prehabilitation intervention can include. First, the baseline assessment of physical function, which required an additional in-person appointment, was not feasible for half of our cohort. Thus, consideration of online screening and baseline assessment is critical for a future prehabilitation trial (https://clinicaltrials.gov/ct2/show/NCT04598087). Current work in Canada is delivering online testing and programming nationally during COVID-19 [57]. Second, delivering a home-based program in the period pre-surgery may be more likely to facilitate adherence to the intervention, removing barriers of additional appointments during this already stressful period. Finally, providing online resources (e.g., movement education) to those with Internet access could be used to further support exercise behavior change pre-surgery. Supported home-based exercise prehabilitation trials have been implemented in other surgical groups [10, 11] with observed improvements in patient outcomes (e.g., improved physical fitness, QOL).

### Examination of changes in PROs and physical function outcomes

As the secondary focus, descriptive analyses of changes in PROs and physical function over three timepoints were examined. Across the surgical timeline (6 weeks), participants experienced a decrease in QOL and health status and an increase in symptom burden, depression, anxiety, and fatigue. Physical function deteriorated during the in-hospital period. While QOL and health status showed improvement in the immediate post-surgery phase (i.e., up to 6 weeks post-surgery), they were still mostly lower than baseline levels. This is in keeping with previous data, indicating that QOL initially deteriorates following surgery [58]. This supports the need for a multiphasic exercise prehabilitation intervention for the HNC surgical patient population, which covers not only pre-surgery but also across the inpatient and into the short-term recovery phases (and pre-adjuvant treatment for a significant percentage of HNC patients [59]). Given the exploratory findings suggesting a negative change in both PROs and physical function, and that for most HNC patients a second “round” of stressors will be faced with adjuvant treatment, there is a need to for future fully powered trials to study the role of exercise across the HNC care timeline (https://clinicaltrials.gov/ct2/show/NCT04598087). This includes ensuring that patients are provided with support at all phases of their disease and treatment trajectory, including the postsurgical inpatient phase as well as into immediate recovery, where outcomes tend to worsen.

### Limitations and future directions

First, while the primary study aim was to assess feasibility, the study was shortened due to the implications of COVID, and thus, even these feasibility findings must be interpreted with caution. Second, the resultant small sample size did not allow for more than the descriptive examination of changes over time in the PROs, physical function, and mobilization assessments to observe trends and report on clinically meaningful changes. Thus, any measure of change in outcome measures must be not taken out of this feasibility context. However, this remains important as we move this work forward to the subsequent phase, an effectiveness-implementation [60] prehabilitation intervention trial (https://clinicaltrials.gov/ct2/show/NCT04598087). Finally, it is also possible that participants were more aware of the importance of movement to their recovery due to their participation in a study, and this may have impacted motivation to move in-hospital and thus changes in outcomes. Future work must continue to examine the patient experience, from PRO and functional outcomes, in order to understand the potential role for exercise in the HNC surgical care pathway.

## Conclusions

This is the first study examining the feasibility of assessment of PROs, physical function, and in-hospital mobilization in the surgical care pathway for HNC patients. An effective, multidisciplinary collaboration between researchers, exercise specialists, and clinicians supported the development of new clinical workflows in HNC surgical care to support the current work. Overall, the results from the present study support that PROs and in-hospital mobilization, tracked with wearable technology, may be feasible and important outcomes to consider in future work examining exercise prehabilitation for HNC surgical patients.

## Data Availability

The datasets generated and/or analyzed during the current study are not publicly available as they contain information that may compromise participant privacy/consent but are available from the corresponding author (JTD) on reasonable request.

## Abbreviations

HNC: Head and neck cancer
PROs: Patient-reported outcomes
ERAS: Enhanced recovery after surgery
QOL: Quality of life
HREBA: Health Research Ethics Board of Alberta
CC: Cancer committee
REDCap: Research Electronic Data Capture
FACT-H&N: Functional Assessment of Cancer Therapy-Head and Neck
EQ-5D VAS: EuroQol visual analogue scale
HADS: Hospital Anxiety and Depression Scale
ESAS-r: Revised Edmonton Symptom Assessment System
FACIT-F: Functional Assessment of Chronic Illness Therapy Fatigue scale
GLTEQ: Godin Leisure-Time Exercise Questionnaire
DXA: Dual-energy X-ray absorptiometry
CPAFLA: Canadian physical activity, fitness & lifestyle approach
CSEP: Canadian Society for Exercise Physiology
6MWT: 6-min walk test
ATS: American Thoracic Society
MCID: Minimum clinically important difference
PA: Physical activity
RT: Resistance training
FT: Flexibility training
BMI: Body mass index

## References

[1] Bray F, Ferlay J, Soerjomataram I, Siegel RL, Torre LA, Jemal A (2018). Global cancer statistics 2018: GLOBOCAN estimates of incidence and mortality worldwide for 36 cancers in 185 countries. CA Cancer J Clin.

[2] Lango MN (2009). Multimodal treatment for head and neck cancer. Surg Clin North Am.

[3] Abendstein H, Nordgren M, Boysen M, Jannert M, Silander E, Ahlner-Elmqvist M, Hammerlid E, Bjordal K (2005). Quality of life and head and neck cancer: a 5 year prospective study. Laryngoscope.

[4] Buchmann L, Conlee J, Hunt J, Agarwal J, White S (2013). Psychosocial distress is prevalent in head and neck cancer patients. Laryngoscope.

[5] Dort JC, Farwell DG, Findlay M, Huber GF, Kerr P, Shea-Budgell MA, Simon C, Uppington J, Zygun D, Ljungqvist O (2017). Optimal perioperative care in major head and neck cancer surgery with free flap reconstruction: a consensus review and recommendations from the enhanced recovery after surgery society. JAMA Otolaryngol Head Neck Surg.

[6] Huber GF, Dort JC (2018). Reducing morbidity and complications after major head and neck cancer surgery: the (future) role of enhanced recovery after surgery protocols. Curr Opin Otolaryngol Head Neck Surg.

[7] Wynter-Blyth V, Moorthy K. Prehabilitation: preparing patients for surgery. BMJ. 2017;358. 10.1136/bmj.j3702 28790033

[8] Carli F, Scheede-Bergdahl C (2015). Prehabilitation to enhance perioperative care. Anesthesiol Clin.

[9] Silver JK, Baima J (2013). Cancer prehabilitation: an opportunity to decrease treatment-related morbidity, increase cancer treatment options, and improve physical and psychological health outcomes. Am J Phys Med Rehabil.

[10] Boereboom C, Doleman B, Lund JN, Williams JP (2016). Systematic review of pre-operative exercise in colorectal cancer patients. Tech Coloproctol.

[11] Ngo-Huang A, Parker NH, Bruera E, Lee RE, Simpson R, O’Connor DP, Petzel MQ, Fontillas RC, Schadler K, Xiao L (2019). Home-based exercise prehabilitation during preoperative treatment for pancreatic cancer is associated with improvement in physical function and quality of life. Integr Cancer Ther.

[12] Cormie P, Atkinson M, Bucci L, Cust A, Eakin E, Hayes S, McCarthy AL, Murnane A, Patchell S, Adams D (2018). Clinical Oncology Society of Australia position statement on exercise in cancer care. Med J Aust.

[13] Capozzi LC, Nishimura KC, McNeely ML, Lau H, Culos-Reed SN (2016). The impact of physical activity on health-related fitness and quality of life for patients with head and neck cancer: a systematic review. Br J Sports Med.

[14] Sandmæl JA, Bye A, Solheim TS, Stene GB, Thorsen L, Kaasa S, Lund JÅ, Oldervoll LM (2017). Feasibility and preliminary effects of resistance training and nutritional supplements during versus after radiotherapy in patients with head and neck cancer: a pilot randomized trial. Cancer.

[15] Cooper CL, Whitehead A, Pottrill E, Julious SA, Walters SJ (2018). Are pilot trials useful for predicting randomisation and attrition rates in definitive studies: a review of publicly funded trials. Clin Trials.

[16] Harris PA, Taylor R, Thielke R, Payne J, Gonzalez N, Conde JG (2009). Research electronic Data Capture (REDCap)—a metadata-driven methodology and workflow process for providing translational research informatics support. J Biom Inform.

[17] Cella DF, Tulsky DS, Gray G, Sarafian B, Linn E, Bonomi A, Silberman M, Yellen SB, Winicour P, Brannon J (1993). The Functional Assessment of Cancer Therapy scale: development and validation of the general measure. J Clin Oncol.

[18] List MA, Ritter-Sterr C, Lansky SB (1990). A performance status scale for head and neck cancer patients. Cancer.

[19] Cracchiolo JR, Klassen AF, Young-Afat DA, Albornoz CR, Cano SJ, Patel SG, Pusic AL, Matros E (2019). Leveraging patient-reported outcomes data to inform oncology clinical decision making: introducing the FACE-Q head and neck cancer module. Cancer.

[20] Rabin R, Fd C (2001). EQ-SD: a measure of health status from the EuroQol Group. Ann Med.

[21] Zigmond AS, Snaith RP (1983). The hospital anxiety and depression scale. Acta Psychiatr Scand.

[22] Katz MR, Kopek N, Waldron J, Devins GM, Tomlinson G (2004). Screening for depression in head and neck cancer. Psycho-Oncology: Journal of the Psychological, Social and Behavioral Dimensions of. Cancer.

[23] Chang VT, Hwang SS, Feuerman M (2000). Validation of the Edmonton symptom assessment scale. Cancer.

[24] Watanabe SM, Nekolaichuk C, Beaumont C, Johnson L, Myers J, Strasser F (2011). A multicenter study comparing two numerical versions of the Edmonton Symptom Assessment System in palliative care patients. J Pain Symptom Manage.

[25] Yellen SB, Cella DF, Webster K, Blendowski C, Kaplan E (1997). Measuring fatigue and other anemia-related symptoms with the Functional Assessment of Cancer Therapy (FACT) measurement system. J Pain Symptom Manage.

[26] Cella D, Lai J-S, Stone A (2011). Self-reported fatigue: one dimension or more? Lessons from the Functional Assessment of Chronic Illness Therapy—Fatigue (FACIT-F) questionnaire. Support Care Cancer.

[27] Godin G, Shephard R (1985). A simple method to assess exercise behavior in the community. Can J Appl Sport Sci.

[28] Campbell KL, Winters-Stone K, Wiskemann J, May AM, Schwartz AL, Courneya KS, Zucker D, Matthews C, Ligibel J, Gerber L (2019). Exercise guidelines for cancer survivors: consensus statement from international multidisciplinary roundtable. Med Sci Sports Exerc.

[29] Wu Y, Wang W, Liu T, Zhang D (2017). Association of grip strength with risk of all-cause mortality, cardiovascular diseases, and cancer in community-dwelling populations: a meta-analysis of prospective cohort studies. J Am Med Dir Assoc.

[30] Bohannon RW, Magasi SR, Bubela DJ, Wang YC, Gershon RC (2012). Grip and knee extension muscle strength reflect a common construct among adults. Muscle Nerve.

[31] Canadian physical activity lifestyle approach: fitness & lifestyle approach (CPAFLA) (2003). CSEP-health & fitness program's health-related appraisal and counselling strategy.

[32] Springer BA, Marin R, Cyhan T, Roberts H, Gill NW (2007). Normative values for the unipedal stance test with eyes open and closed. J Geriatr Phys Ther.

[33] Canadian Society for Exercise Physiology: Canadian Society for Exercise Physiology-Physical Activity Training for Health (CSEP-PATH): Canadian Society for Exercise Physiology; 2013.

[34] Capozzi LC, McNeely ML, Lau HY, Reimer RA, Giese-Davis J, Fung TS, Culos-Reed SN (2016). Patient-reported outcomes, body composition, and nutrition status in patients with head and neck cancer: Results from an exploratory randomized controlled exercise trial. Cancer.

[35] Eden MM, Tompkins J, Verheijde JL (2018). Reliability and a correlational analysis of the 6MWT, ten-meter walk test, thirty second sit to stand, and the linear analog scale of function in patients with head and neck cancer. Physiother Theory Pract.

[36] Solway S, Brooks D, Lacasse Y, Thomas S (2001). A qualitative systematic overview of the measurement properties of functional walk tests used in the cardiorespiratory domain. Chest.

[37] ATS Committee on Proficiency Standards for Clinical Pulmonary Function Laboratories (2002). ATS statement: guidelines for the six-minute walk test. Am J Respir Crit Care Med.

[38] Lynch BM, Nguyen NH, Moore MM, Reeves MM, Rosenberg DE, Boyle T, Milton S, Friedenreich CM, Vallance JK, English DR (2019). Maintenance of physical activity and sedentary behavior change, and physical activity and sedentary behavior change after an abridged intervention: secondary outcomes from the ACTIVATE trial. Cancer.

[39] Tackney MS, Cook DG, Stahl D, Ismail K, Williamson E, Carpenter J (2021). A framework for handling missing accelerometer outcome data in trials. Trials.

[40] Coretti S, Ruggeri M, McNamee P (2014). The minimum clinically important difference for EQ-5D index: a critical review. Expert Rev Pharmacoecon Outcomes Res.

[41] Ringash J, Bezjak A, O'sullivan B, Redelmeier DA (2004). Interpreting differences in quality of life: the FACT-H&N in laryngeal cancer patients. Qual Life Res.

[42] Pickard AS, Neary MP, Cella D (2007). Estimation of minimally important differences in EQ-5D utility and VAS scores in cancer. Health Qual Life Outcomes.

[43] Puhan MA, Frey M, Büchi S, Schünemann HJ (2008). The minimal important difference of the hospital anxiety and depression scale in patients with chronic obstructive pulmonary disease. Health Qual Life Outcomes.

[44] Hui D, Shamieh O, Paiva CE, Khamash O, Perez-Cruz PE, Kwon JH, Muckaden MA, Park M, Arthur J, Bruera E (2016). Minimal clinically important difference in the physical, emotional, and total symptom distress scores of the Edmonton Symptom Assessment System. J Pain Symptom Manage.

[45] Warkentin LM, Majumdar SR, Johnson JA, Agborsangaya CB, Rueda-Clausen CF, Sharma AM, Klarenbach SW, Karmali S, Birch DW, Padwal RS (2014). Weight loss required by the severely obese to achieve clinically important differences in health-related quality of life: two-year prospective cohort study. BMC Med.

[46] Bobos P, Nazari G, Lu S, MacDermid JC (2019). Measurement properties of the hand grip strength assessment. a systematic review with meta-analysis. Archives of physical medicine and rehabilitation.

[47] Goldberg A, Casby A, Wasielewski M (2011). Minimum detectable change for single-leg-stance-time in older adults. Gait Posture.

[48] McNeely ML, Sellar C, Williamson T, Shea-Budgell M, Joy AA, Lau HY, Easaw JC, Murtha AD, Vallance J, Courneya K (2019). Community-based exercise for health promotion and secondary cancer prevention in Canada: protocol for a hybrid effectiveness-implementation study. BMJ open.

[49] Bohannon RW, Crouch R (2017). Minimal clinically important difference for change in 6-minute walk test distance of adults with pathology: a systematic review. J Eval Clin Pract.

[50] Brahmbhatt P, Sabiston CM, Lopez C, Chang E, Goodman J, Jones J, McCready D, Randall I, Rotstein S, Santa Mina D: Feasibility of prehabilitation prior to breast cancer surgery: a mixed-methods study. Front Oncol 2020:1979. 10.3389/fonc.2020.571091 PMC7544900 33072603

[51] Ester M, Culos-Reed SN, Abdul-Razzak A, Daun JT, Duchek D, Francis G, Bebb G, Black J, Arlain A, Gillis C (2021). Feasibility of a multimodal exercise, nutrition, and palliative care intervention in advanced lung cancer. BMC Cancer.

[52] Daun JT, Capozzi LC, Roldan-Urgoiti G, McDonough MH, Easaw JC, McNeely M, Francis GJ, Williamson T, Danyluk J, McLaughlin M et al: ACE-Neuro: a tailored exercise oncology program for neuro-oncology patients – study protocol. MedRxiv (Under Review at Contemporary Clinical Trials Communications) 2022. 10.1016/j.conctc.2022.100925 PMC9198374 35720248

[53] Chang SS (2020). The patient perspective—a valuable but untapped resource in otolaryngology–head and neck surgery. JAMA Otolaryngol Head Neck Surg.

[54] Gresham G, Schrack J, Gresham LM, Shinde AM, Hendifar AE, Tuli R, Rimel B, Figlin R, Meinert CL, Piantadosi S (2018). Wearable activity monitors in oncology trials: current use of an emerging technology. Contemp Clin Trials.

[55] Wolk S, Meißner T, Linke S, Müssle B, Wierick A, Bogner A, Sturm D, Rahbari NN, Distler M, Weitz J (2017). Use of activity tracking in major visceral surgery—the enhanced perioperative mobilization (EPM) trial: study protocol for a randomized controlled trial. Trials.

[56] Demark-Wahnefried W, Aziz NM, Rowland JH, Pinto BM (2005). Riding the crest of the teachable moment: promoting long-term health after the diagnosis of cancer. J Clin Oncol.

[57] Culos-Reed SN (2020). Dissemination, implementation, and effectiveness of the exercise oncology survivorship partnership model: reaching rural cancer survivors to enhance quality of life. CIHR, CCS, ACF. $2.5m, 2020-25.

[58] Dropkin MJ (1999). Body image and quality of life after head and neck cancer surgery. Cancer Pract.

[59] Anderson G, Ebadi M, Vo K, Novak J, Govindarajan A, Amini A (2021). An updated review on head and neck cancer treatment with radiation therapy. Cancers.

[60] Curran GM, Bauer M, Mittman B, Pyne JM, Stetler C (2012). Effectiveness-implementation hybrid designs: combining elements of clinical effectiveness and implementation research to enhance public health impact. Med Care.

